# Epidemiological characteristics of leptospirosis and serological surveillance among reservoir animals and healthy populations in Fujian, China, 2004–2024

**DOI:** 10.3389/fpubh.2026.1891639

**Published:** 2026-08-24

**Authors:** Guoying Xu, Yufeng Li, Weijun Liu, Tengwei Han, Zhiwei Zeng, Lingqiong Huang, Fangqing He, Yuwei Nian, Shiju Hu, Fen Lin, Shenggen Wu, Fangzhen Xiao

**Affiliations:** 1 Fujian Provincial Key Laboratory of Zoonosis Research, Fujian Center for Disease Control and Prevention, Fuzhou, China; 2 The School of Public Health, Fujian Medical University, Fuzhou, China

**Keywords:** epidemiological characteristics, host animals, leptospirosis, spatial autocorrelation, spatiotemporal clustering

## Abstract

**Background:**

Leptospirosis is a globally distributed zoonosis caused by pathogenic *Leptospira*. This study investigated the epidemiological characteristics of leptospirosis in Fujian Province, China, from 2004 to 2024 to provide evidence for risk assessment and prevention strategies.

**Methods:**

Data on human leptospirosis cases, reservoir animals, and healthy populations in Fujian Province from 2004 to 2024 were collected through the Infectious Disease Surveillance System of the Chinese Center for Disease Control and Prevention and 31 surveillance sites. Descriptive epidemiological methods were used to analyze temporal, spatial, and population distribution characteristics. Joinpoint regression, spatial autocorrelation, and spatiotemporal scan analyses were performed to identify temporal trends and spatiotemporal clustering. Serological data from rodents and healthy populations were analyzed to assess antibody prevalence and serogroup distribution.

**Results:**

From 2004 to 2024, the incidence of leptospirosis in Fujian Province showed a significant downward trend, with an average annual incidence of 0.08 cases per 100,000 population. A total of 681 cases were reported, mainly between July and October and concentrated in northern and north-central Fujian. Farmers and individuals aged 30–69 years constituted the major affected populations, and the age at onset gradually increased over time. Significant spatial clustering was observed (Moran’s *I* > 0, *p* < 0.05), with Songxi County identified as a hotspot and southern Fujian as a coldspot. Spatiotemporal analysis further identified Wuping County as the primary spatiotemporal cluster during June 2005 to August 2014. From 2005 to 2024, the average rodent capture rate at surveillance sites was 6.75%, predominantly involving *Rattus losea* and *Rattus tanezumi*. The average seroprevalence of *Leptospira* antibodies was 32.89% in rodents and 17.81% in healthy individuals. Javanica, Autumnalis, and Pomona predominated in rodents, whereas Autumnalis, Grippotyphosa, and Australis predominated in healthy individuals.

**Conclusion:**

Leptospirosis incidence in Fujian Province declined substantially from 2004 to 2024 but remained characterized by marked spatial clustering and seasonal patterns. Rodents remained important reservoir hosts of *Leptospira*, while serogroup distributions differed between reservoir hosts and humans, indicating complex transmission dynamics. Long-term surveillance provides valuable information for understanding the epidemiology of leptospirosis and supporting prevention and control efforts.

## 1 Introduction

Leptospirosis is a zoonotic infectious disease caused by pathogenic *Leptospira*, primarily prevalent in tropical and subtropical regions (1). To date, at least 200 animal species worldwide have been identified as natural reservoirs of pathogenic *Leptospira*. Rodents, in particular, are recognized as the primary reservoirs and major sources of human infection (2). The disease is primarily transmitted through direct or indirect contact with soil or water contaminated by infected animal urine (3), while human-to-human transmission remains extremely rare. Despite the rarity of person-to-person transmission, human infection remains highly prevalent due to exposure to infected animal reservoirs and contaminated environmental sources. Leptospirosis infects over one million people annually, resulting in nearly 60,000 deaths and imposing a significant global public health burden (4). Its clinical manifestations are diverse, ranging from mild acute febrile illness to severe hepatic and renal failure, hemorrhagic disorders, and even death (5).

In China, leptospirosis has been classified as a Category B infectious disease since 1955. To date, 29 of China’s 34 provinces have reported over 2.5 million cases of leptospirosis, resulting in more than 200,000 deaths (6). Since the early 21st century, national incidence and mortality rates have declined due to rodent control, improved sanitation, and enhanced vaccination and treatment strategies (7). The disease is currently maintained at a low endemic level nationwide. In Fujian Province, where the first case was recorded in 1956, the epidemic expanded continuously with periodic outbreaks, peaking between 1972 and 1973. The epidemic began to subside in the late 1970s. In the 21st century, the disease has generally remained at low levels, though sporadic cases and localized outbreaks continue to occur. Fujian remains a key endemic area for leptospirosis, posing an ongoing threat to public health, therefore making surveillance and prevention efforts crucial.

Fujian Province, located in a subtropical region with a warm and humid climate, offers an ideal habitat for reservoir animals such as rodents, due to its high temperatures, humidity, and abundant water resources. These environmental conditions contribute to the province’s status as a highly endemic region for leptospirosis. As a result, Fujian has established a long-term surveillance program for anti-*Leptospira* antibodies in reservoir animals and healthy human populations, with a focus on rodent capture density, species composition, seropositivity rates, and predominant serogroups. This study analyzes changes in the epidemiological characteristics and spatio-temporal clustering patterns of leptospirosis in Fujian Province from 2004 to 2024. It also examines shifts in antibody levels and serogroup composition among reservoir animals and healthy populations, aiming to provide scientific evidence for the formulation of disease control strategies and the refinement of surveillance protocols.

## 2 Methods

### 2.1 Study sites

Fujian Province is located on the southeastern coast of China, spanning latitudes 23°31′–28°18′N and longitudes 115°50′–120°43′E. It borders Zhejiang Province to the northeast, Jiangxi Province to the northwest, and Guangdong Province to the southwest, and faces Taiwan across the Taiwan Strait to the southeast. The province’s terrain is characterized by higher elevations in the northwest and lower elevations in the southeast, forming a landscape dominated by mountainous and hilly regions. Mountains and hills account for approximately 90% of the total land area. Fujian has a subtropical monsoon climate. The province covers a land area of 124,000 km^2^ and a maritime area of 136,000 km^2^. Administratively, Fujian comprises nine prefecture-level cities and the Pingtan Comprehensive Experimental Zone, further divided into 84 county-level administrative units. According to official statistics (8), Fujian Province had a resident population of approximately 41.93 million at the end of 2024, with an urbanization rate of 71.8%. Although Fujian has undergone rapid urbanization, agricultural activities, including crop production and livestock farming, remain widespread in rural areas (9).

### 2.2 Data collection

This study integrates data from human leptospirosis surveillance, host animal monitoring, and healthy population surveillance. Human case records and population data for leptospirosis in Fujian Province from 2004 to 2024 were obtained from the Infectious Disease Reporting System of the Chinese Center for Disease Control and Prevention. These data included annual county-level case counts and detailed demographic and epidemiological information, including residential address, date of symptom onset, date of death, sex, age, and occupation. Data on host animal surveillance and healthy population monitoring were obtained from the annual Leptospirosis Surveillance Program of Fujian Province.

### 2.3 Case definition

According to the health industry standard of the People’s Republic of China for the diagnostic criteria of leptospirosis, leptospirosis cases were classified as suspected cases, clinically diagnosed cases, and laboratory-confirmed cases. (1) Suspected cases are defined as individuals who, within 1–30 days prior to disease onset, had a history of contact with contaminated water, urine from infected animals, or blood from infected animals, and who present with clinical symptoms such as fever, myalgia, or arthralgia. (2) Clinically diagnosed cases are suspected cases accompanied by conjunctival suffusion, gastrocnemius muscle tenderness, or lymphadenopathy. (3) Laboratory-confirmed cases are suspected cases that meet at least one of the following criteria: isolation of spirochetes from blood, cerebrospinal fluid, or urine; detection of spirochetal nucleic acid; a fourfold or greater increase in serum antibody titers between acute- and convalescent-phase samples; or a single serum antibody titer ≥1:400 (10). In this study, only clinically diagnosed cases and laboratory-confirmed cases were included in the analysis.

Serological testing was performed using the Microscopic Agglutination Test (MAT). Reference strains of *Leptospira* were provided by the National Center for Disease Control and Prevention, Chinese Center for Disease Control and Prevention. The MAT positivity thresholds were defined as antibody titers ≥1:50 for healthy human sera, ≥1:400 for patient sera, and ≥1:20 for animal sera. When antibodies against multiple serogroups were detected in a single sample, the serogroup with the highest antibody titer was considered the infecting serogroup (10, 11).

### 2.4 Host animal and healthy population surveillance

Since 2005, the Fujian Provincial Center for Disease Control and Prevention has established long-term sentinel surveillance sites in Changtai District and Wuping County in accordance with the Fujian Provincial Leptospirosis Surveillance Program to conduct reservoir host surveillance for *Leptospira* and serological surveillance of healthy populations. Surveillance in Changtai District was conducted from 2005 to 2018, while surveillance in Wuping County has been conducted from 2011 to 2024, with data unavailable for 2013. In addition, since 2011, two to three counties or districts have been randomly selected each year across the province to conduct reservoir host surveillance and serological surveillance of healthy populations. At each surveillance site, at least 300 rodent traps were deployed annually, with trapping conducted mainly between April and October in natural environments. Standardized trapping protocols were applied consistently throughout the surveillance period. Laboratory testing was performed according to standardized national and provincial surveillance protocols using reference strains provided by the Chinese Center for Disease Control and Prevention. Internal quality control procedures were implemented throughout the surveillance period, and laboratory personnel received standardized training and followed standardized operating procedures.

### 2.5 Data analysis

Joinpoint Regression Program was used to analyze temporal trends in leptospirosis incidence from 2004 to 2024, with the annual percentage change (APC) calculated to quantify the magnitude of the trend. ArcGIS version 10.3.1 was used for map production. Descriptive epidemiological methods were applied to characterize the distribution of leptospirosis by time, place, and population, including sex, age, and occupation. The Kruskal–Wallis test was used to compare differences in incidence rates among counties across four defined periods, as well as annual variations in the median age of cases.

Global and local spatial autocorrelation analyses were conducted using ArcGIS 10.3.1 to evaluate spatial dependence, identify clustering patterns, and detect spatial hotspots and outliers. Spatiotemporal cluster analysis was performed using SaTScan version 10.1, with log-likelihood ratio (LLR) and relative risk (RR) calculated for each cluster. A significance level of *p* < 0.05 was considered statistically significant, indicating the presence of spatiotemporal clustering. Results were visualized using ArcGIS 10.3.1. Clusters with the highest LLR values were defined as primary clusters, while other statistically significant clusters were classified as secondary clusters.

Chi-square tests were used to compare relative rodent densities and seropositivity rates among animal hosts and human populations. Chi-square trend tests were applied to assess long-term trends in *Leptospira* seropositivity among rodents and healthy populations. Fisher′s exact test was used to compare the distribution of *Leptospira* serogroups. Spearman′s rank correlation analysis was conducted to examine associations between leptospirosis incidence at surveillance sites, animal seropositivity rates, and human seropositivity rates. All statistical analyses were performed using R software (version 4.5.0). A significance level of *p* < 0.05 was applied to all analyses.

## 3 Results

### 3.1 Epidemiological feature

**Figure 1:**
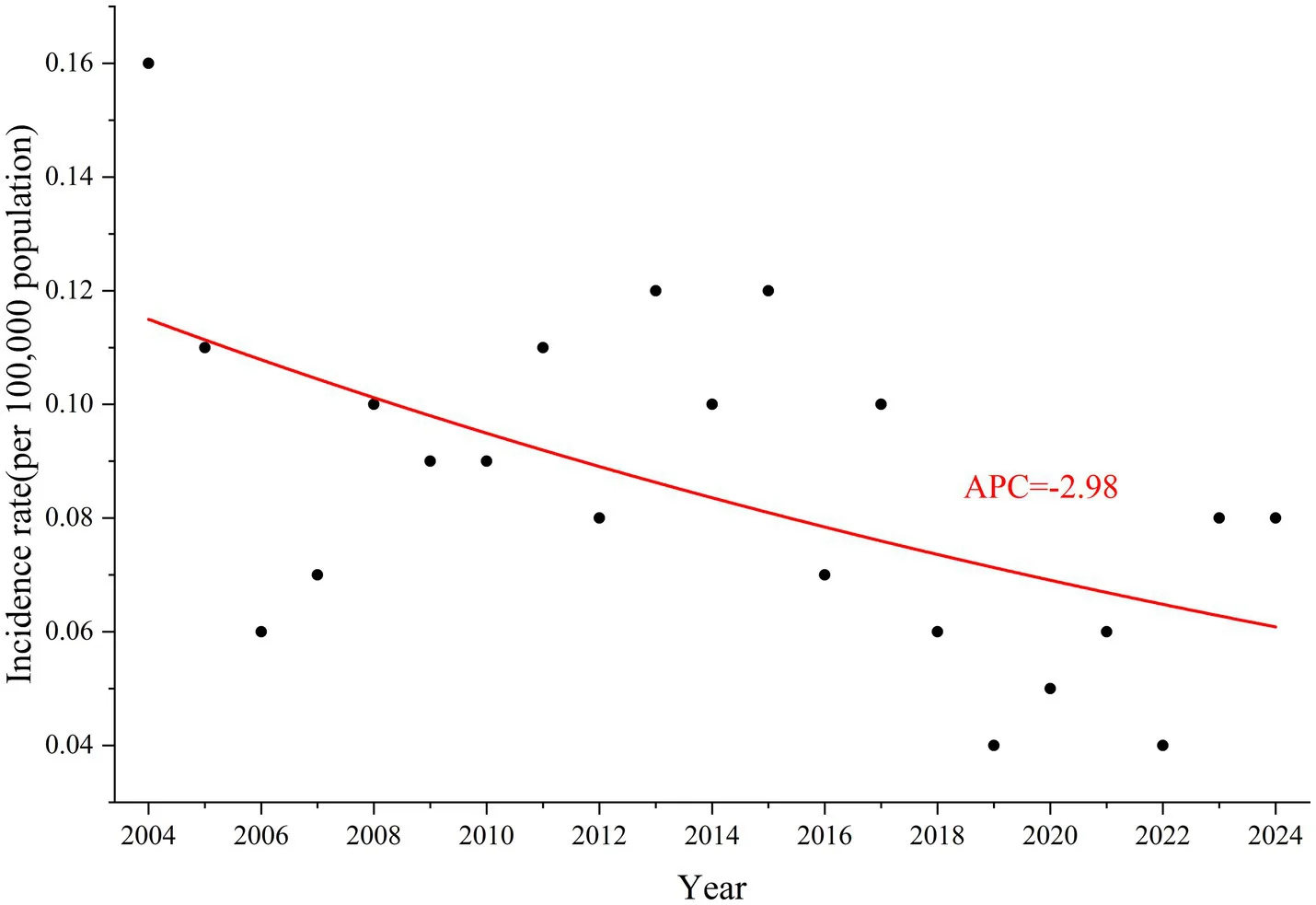
The incidence rate of leptospirosis in Fujian Province, 2004–2024.

From 2004 to 2024, a total of 681 leptospirosis cases, including 4 deaths, were reported in Fujian Province. The average annual incidence rate was 0.08 cases per 100,000 population, the mortality rate was 0.001 cases per 100,000 population, and the case-fatality rate was 0.59%. The incidence rate declined from 2004 to 2006, followed by a gradual increase beginning in 2007. After reaching a relatively high level in 2013, the incidence rate declined again, with a slight rebound observed during 2023–2024. The highest incidence rate was recorded in 2004 (0.16 cases per 100,000 population), while the lowest occurred in 2019 (0.04 cases per 100,000 population). Compared with 2004, the incidence rate in 2024 decreased by 47.78%. Joinpoint regression analysis demonstrated that the annual incidence rate of leptospirosis in Fujian Province exhibited an overall decreasing trend over the 21-year study period (APC = −2.98, 95% CI: −5.850 to −0.150, *p* < 0.05) (Figure 1).

#### 3.1.1 Regional distribution of leptospirosis

**Figure 2:**
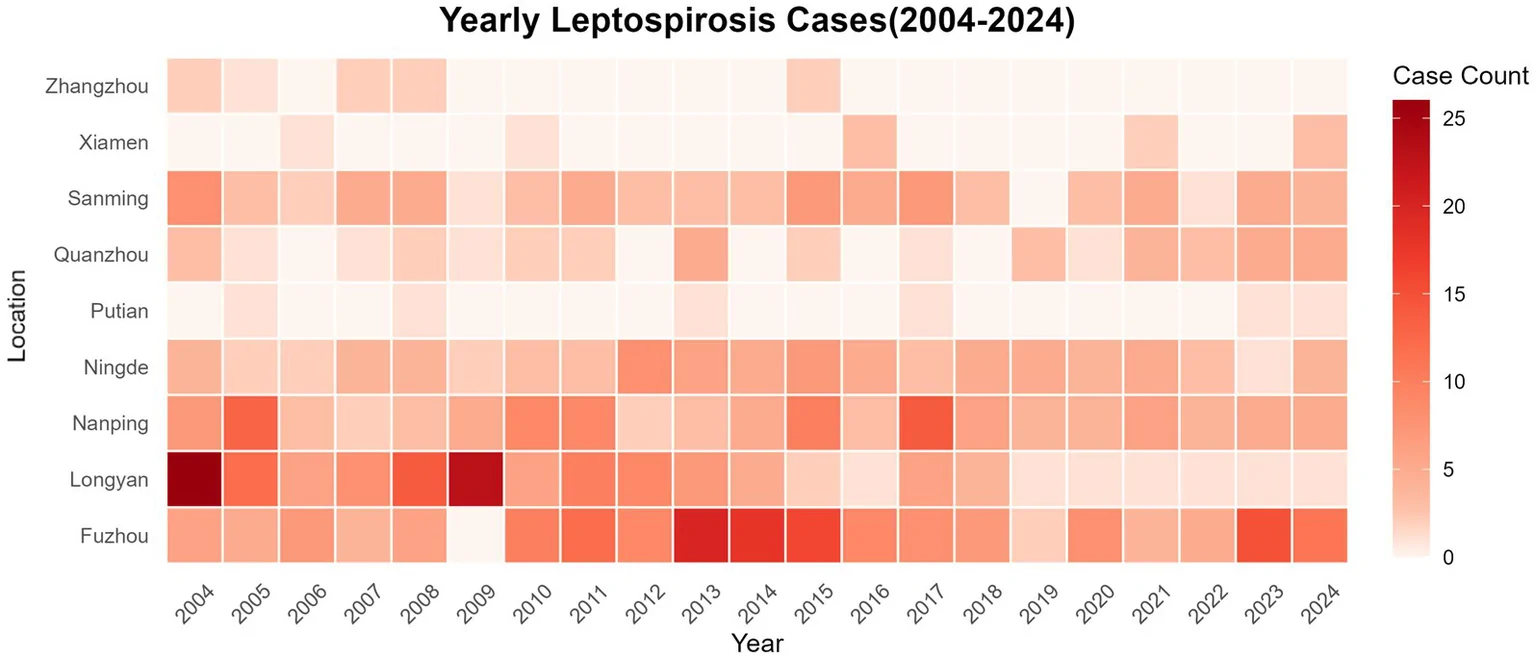
Spatial distribution of leptospirosis cases by city in Fujian Province, 2004–2024.

From 2004 to 2024, all nine prefecture-level cities in Fujian Province reported cases of leptospirosis. In terms of case numbers, Fuzhou reported the highest number of cases (*n* = 182), followed by Longyan (*n* = 145) and Nanping (*n* = 122). Ningde (*n* = 85), Sanming (*n* = 81), and Quanzhou (*n* = 41) reported a moderate number of cases, whereas Xiamen, Zhangzhou, and Putian reported relatively few cases, with a combined total of 25 cases (Figure 2). With respect to incidence rates, Longyan exhibited the highest incidence during 2004–2012, followed by Nanping and Fuzhou. After 2013, leptospirosis cases were predominantly concentrated in northern and north-central Fujian, while incidence rates in other regions remained comparatively low (Supplementary Figure S1).

**Figure 3:**
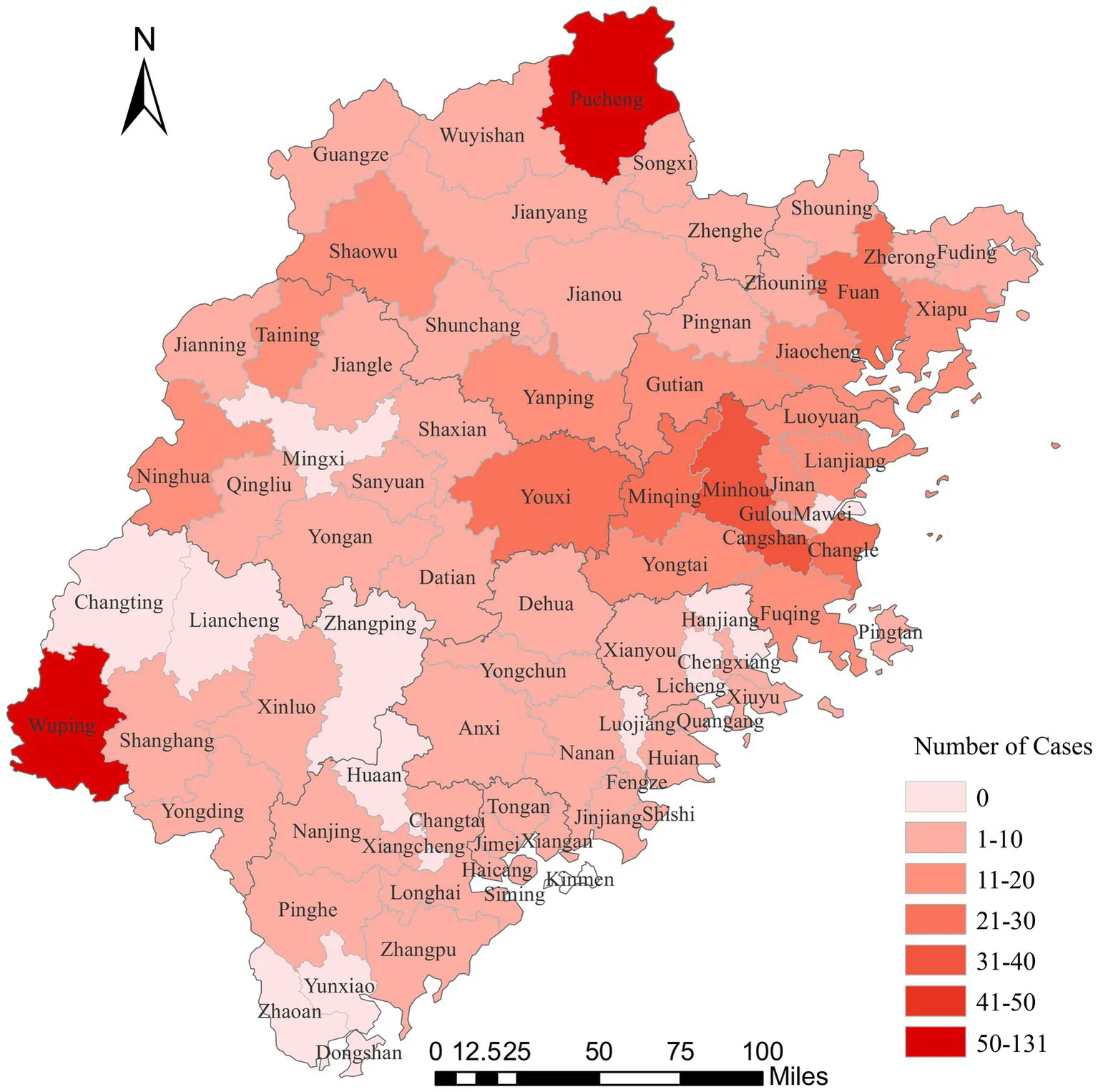
Number of leptospirosis cases by county in Fujian Province, 2004–2024.

**Figure 4:**
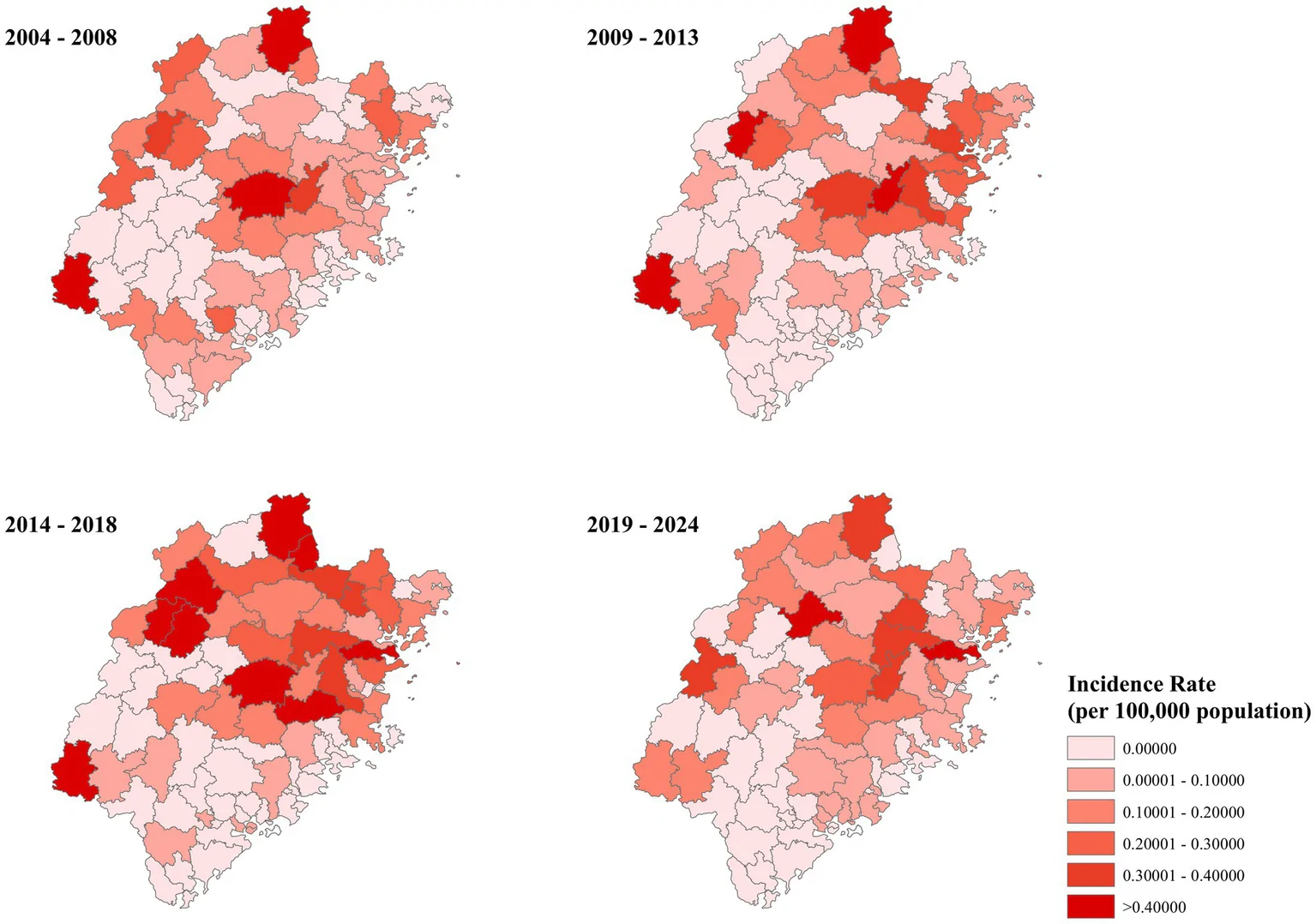
Comparison of leptospirosis incidence rates by stage across counties in Fujian Province, 2004–2024.

Among the 83 counties in Fujian Province (excluding Kinmen County), 70 reported cases of leptospirosis. Wuping County recorded the highest average incidence rate (1.94 cases per 100,000 population), accounting for 19.24% of the total reported cases. Pucheng County ranked second, with an average incidence rate of 0.74 cases per 100,000 population and 51 reported cases (7.49% of the total). In contrast, 48 counties reported fewer than 10 cumulative cases (Figure 3). Analysis of county-level incidence rates across four time periods (2004–2008, 2009–2013, 2014–2018, and 2019–2024) showed distinct spatiotemporal variations. From 2004 to 2008, high-incidence areas were mainly concentrated in Wuping, Pucheng, and Youxi counties. During 2009–2013, high-incidence areas shifted to Wuping, Pucheng, Taining, and Minqing counties. Beginning in 2014, the number of high-incidence counties increased, predominantly in Wuping County and northern Fujian. During 2019–2024, the number of high-incidence counties declined, accompanied by an overall reduction in incidence rates; however, northern and north-central Fujian remained the primary affected regions (Figure 4).

#### 3.1.2 Seasonal distribution of leptospirosis

**Figure 5:**
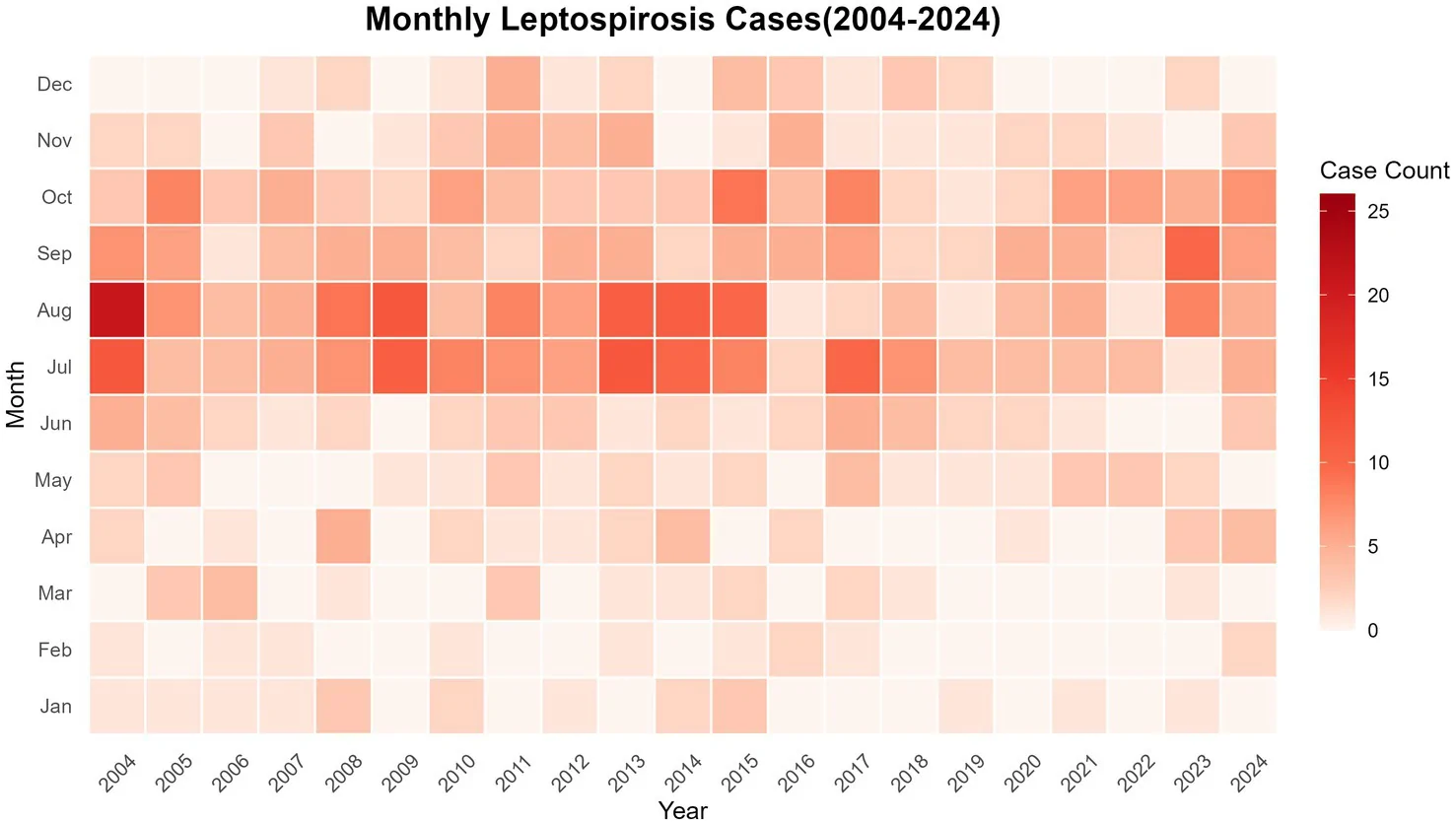
Heatmap of monthly leptospirosis cases in Fujian Province, 2004–2024.

Leptospirosis incidence in Fujian Province exhibited clear seasonal patterns from 2004 to 2024. Cases were predominantly reported between July and October, with cases reported in each of the 21 study years during this period. This peak season accounted for 67.55% (460/681) of all reported cases (Figure 5). In contrast, the period from December to April of the following year represented a low-incidence phase, contributing only 15.12% (103/681) of the total cases. Notably, during this low-incidence period, no cases were reported in certain months over multiple consecutive years.

#### 3.1.3 Population distribution of leptospirosis

**Figure 6:**
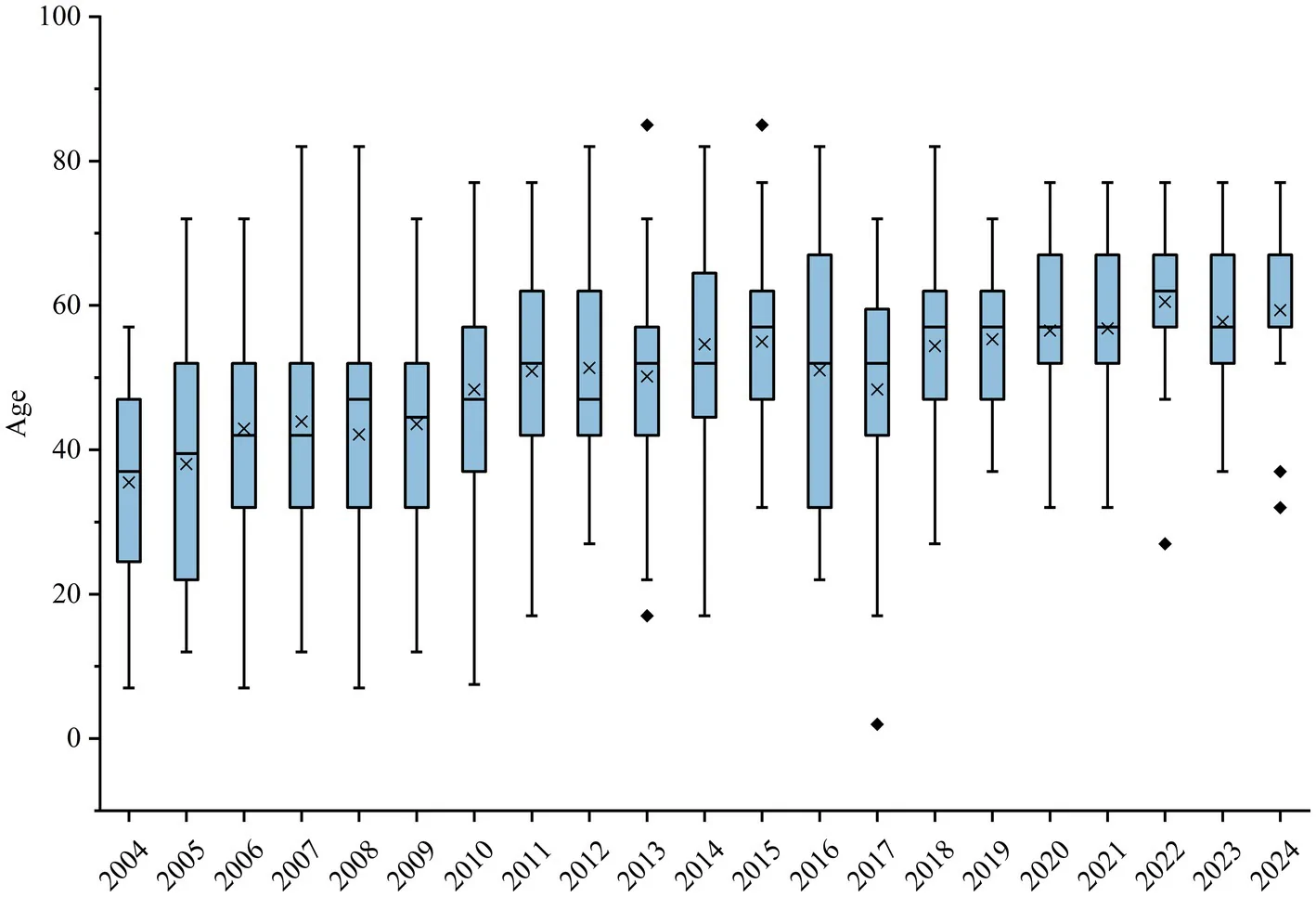
Temporal trend in the median age of leptospirosis cases in Fujian Province, 2004–2024.

**Figure 7:**
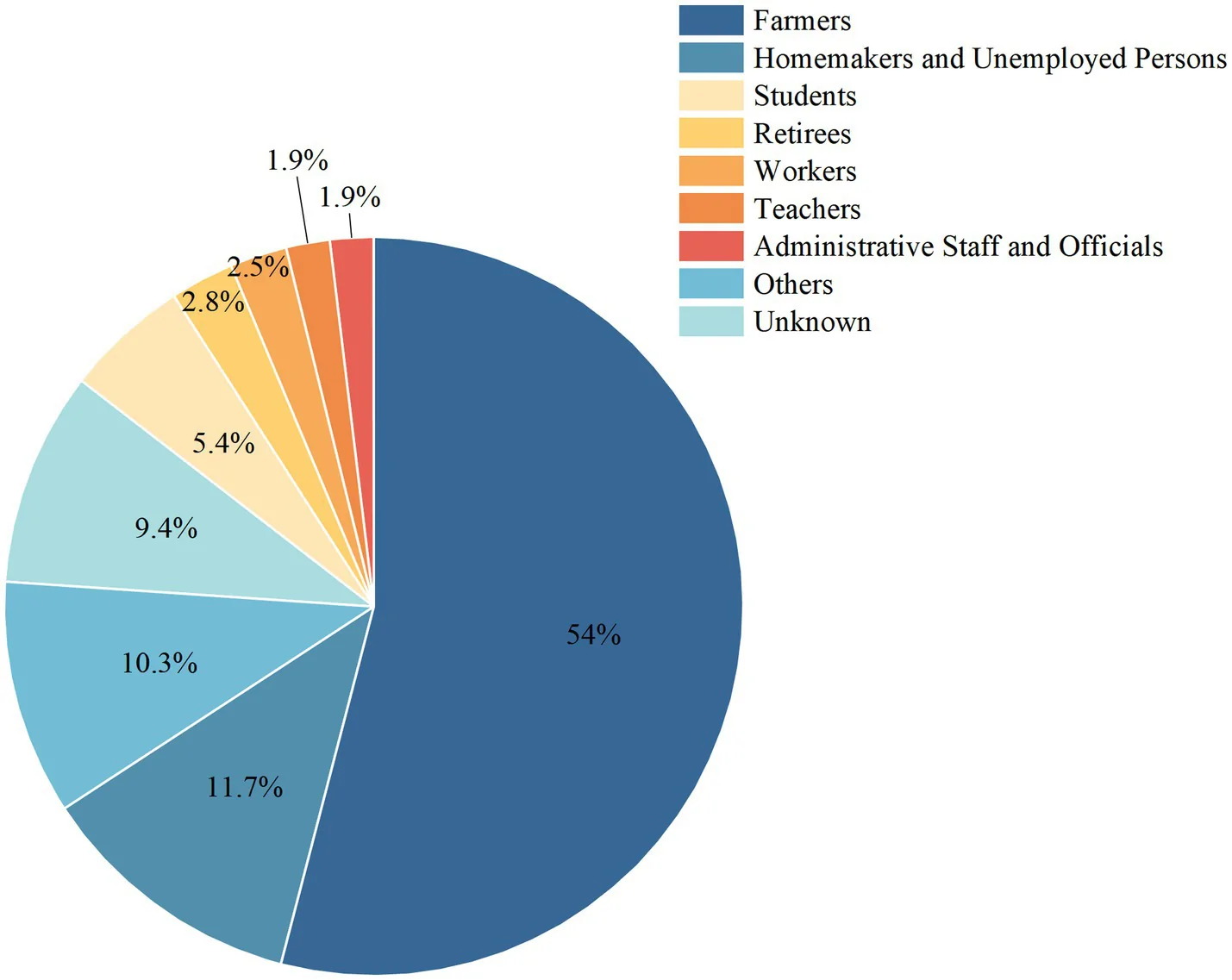
Occupational distribution of leptospirosis cases in Fujian Province, 2004–2024.

In Fujian Province, leptospirosis predominantly affected males, with a male-to-female ratio of 2.76:1 (500/181). The age distribution was broad but concentrated primarily among individuals aged 30–69 years, who accounted for 85.92% (585/681) of all cases. Incidence increased from age 30, peaked in the 50–60-year age group, and subsequently declined. Between 2004 and 2024, the median age of patients increased from 37 to 57 years. The median age was lowest in 2004 (37 years) and highest in 2022 (62 years) (Figure 6), indicating a sustained upward trend in age at onset. Kruskal–Wallis tests of annual age distributions demonstrated statistically significant differences across years (*H* = 132.33, *p* < 0.005). Occupational distribution showed that farmers accounted for the majority of cases (54.04%, 368/681), followed by homemakers and unemployed individuals (11.75%, 80/681) (Figure 7).

### 3.2 Spatial autocorrelation analyses

A global spatial autocorrelation analysis of leptospirosis incidence in Fujian Province from 2004 to 2024 showed a positive Moran’s *I* value (Moran’s *I* = 0.11, Z = 2.14, *p* = 0.03), indicating statistically significant spatial clustering of leptospirosis cases during the study period.

**Figure 8:**
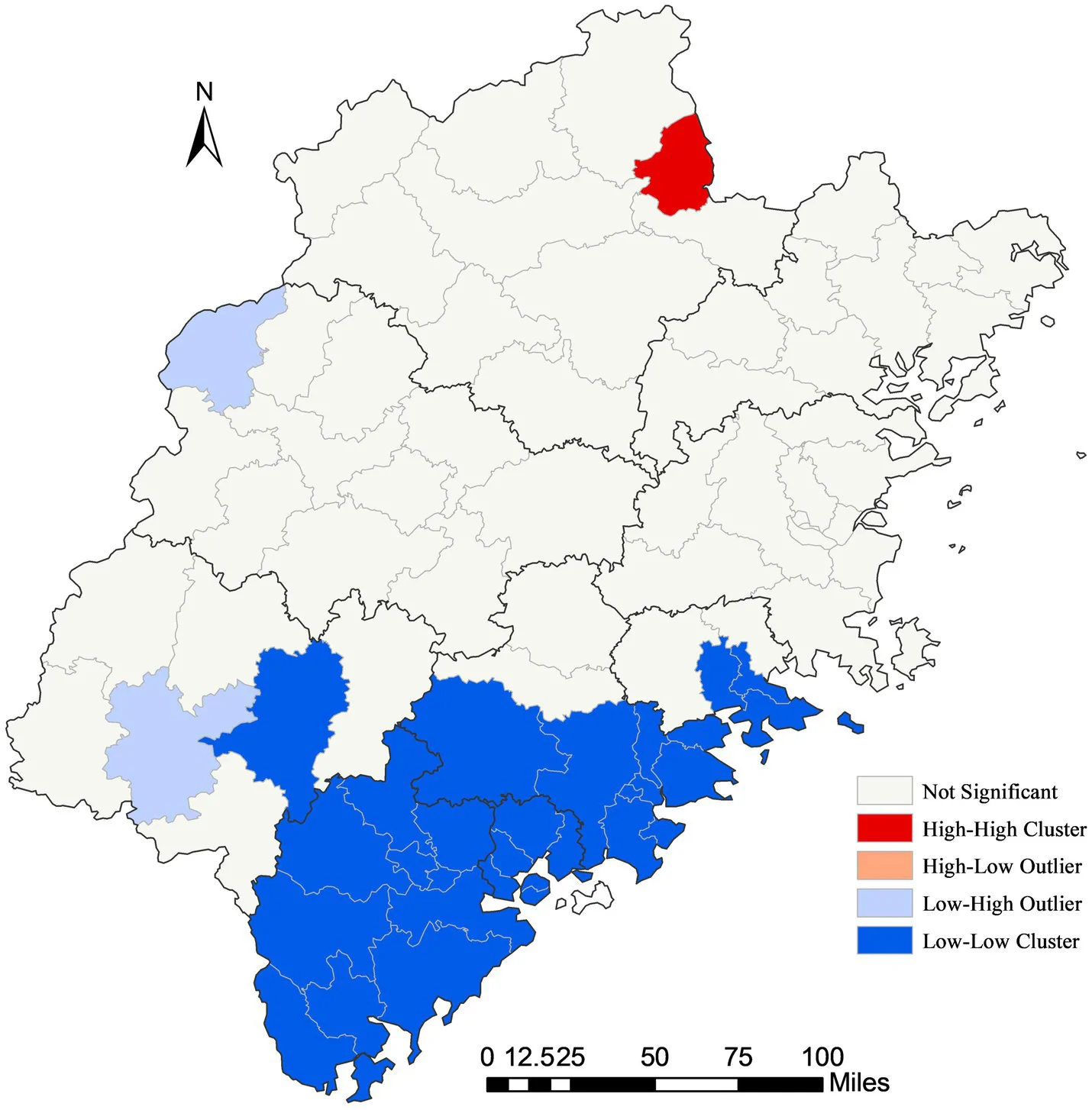
Local spatial autocorrelation analysis of leptospirosis in Fujian Province, 2004 to 2024.

To further identify localized clustering patterns, a local spatial autocorrelation analysis was conducted on county-level incidence rates from 2004 to 2024, and a cluster distribution map was generated. The analysis identified one high–high cluster in Songxi County, whereas low–low clusters were primarily concentrated in southern Fujian, mainly encompassing parts of Zhangzhou, Xiamen, Quanzhou, and Putian (Figure 8).

### 3.3 Spatiotemporal scan analysis

**Figure 9:**
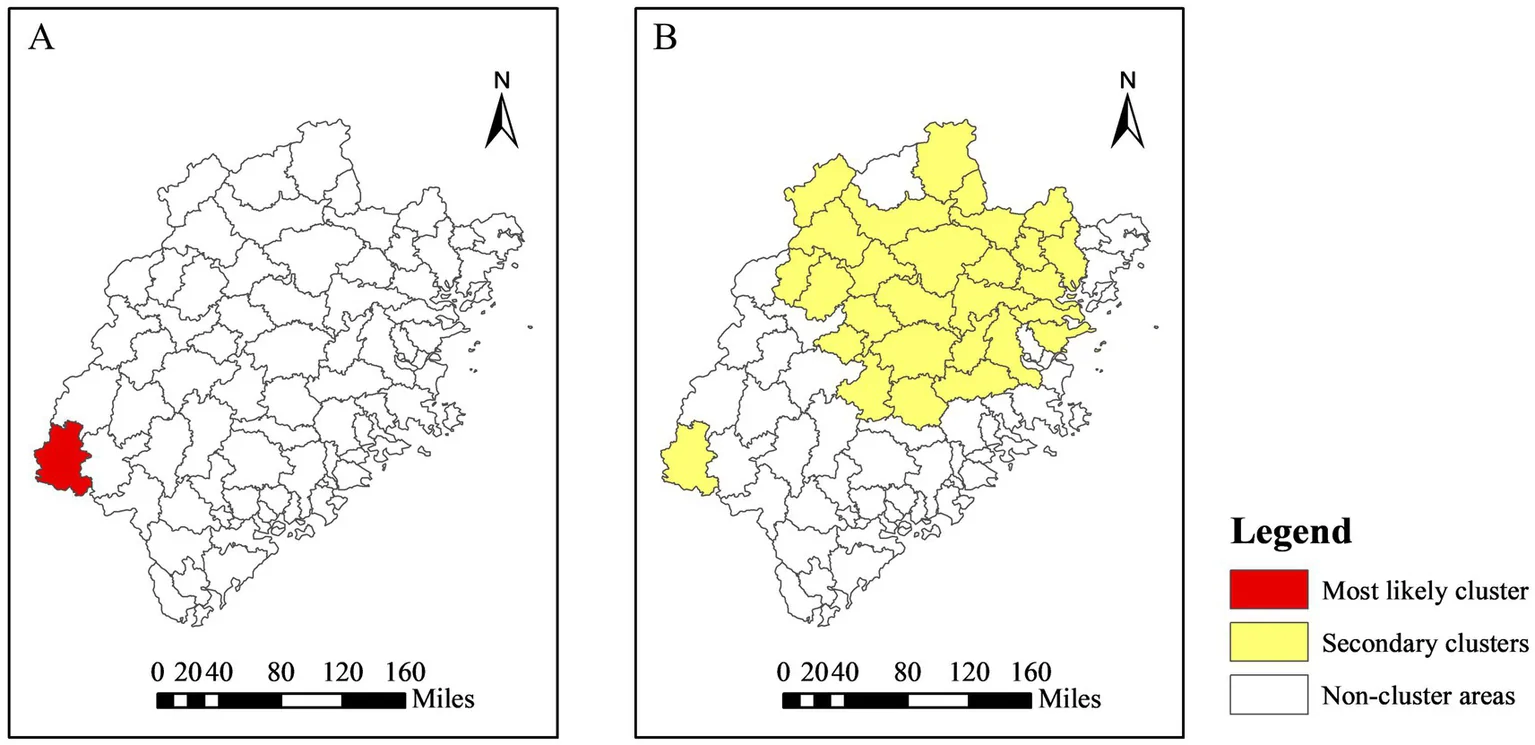
Spatiotemporal clusters of leptospirosis cases in Fujian Province, 2005 to 2024. **(A)** The most likely cluster; **(B)** the secondary clusters.

Due to incomplete individual case information in 2004, spatiotemporal scan analysis was conducted using data from 2005 to 2024. The analysis identified the most likely cluster in Wuping County (RR = 46.09, LLR = 260.62, *p* < 0.001), with a temporal window extending from June 2005 to August 2014 (Table 1). In addition, secondary clusters encompassed 28 counties, predominantly distributed across northern and north-central Fujian Province (Table 1 and Figure 9).

**Table 1:** The cluster results of space–time scan for leptospirosis in Fujian Province, 2005 to 2024.

Aggregate type	Counties covered	Aggregation time	Observed cases	Expected cases	RR	LLR	*P*
Type I	1	2005/6–2014/8	94	2.39	46.09	260.62	<0.001
Type II	14	2010/7–2019/9	118	35.75	3.84	64.68	<0.001
	1	2005/6–2015/3	29	2.53	11.99	44.88	<0.001
	1	2017/7–2017/8	6	0.04	157.37	24.36	<0.001
	5	2015/7–2017/10	16	1.86	8.80	20.45	<0.001
	10	2020/4–2024/11	34	11.83	2.98	14.14	0.023

### 3.4 Host animal and healthy population surveillance

#### 3.4.1 Host animal surveillance

**Figure 10:**
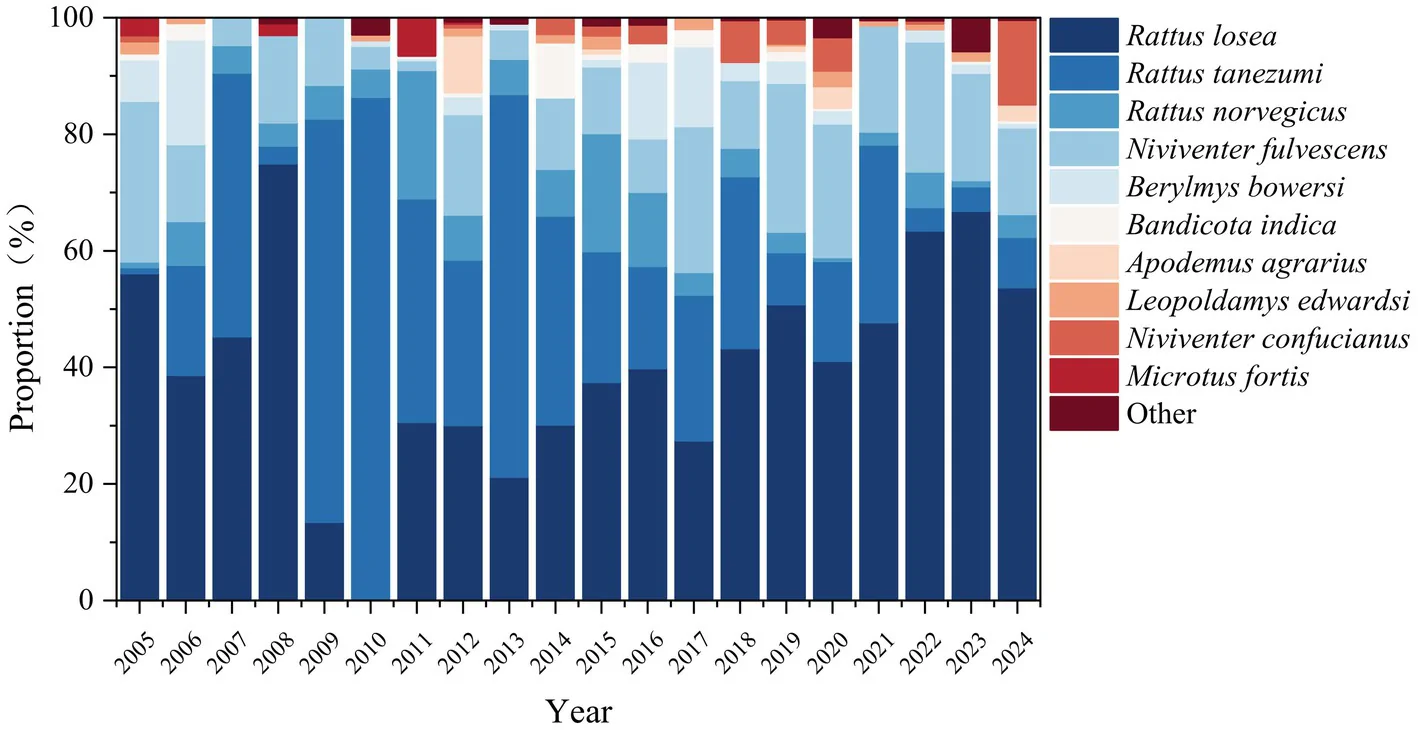
Species composition of animals at leptospirosis surveillance sites in Fujian Province, 2005–2024.

Antibody surveillance among reservoir animals and human populations was conducted in 31 counties of Fujian Province, comprising 57 monitoring rounds from 2005 to 2024. A total of 56,979 rodent traps were deployed, yielding 3,847 captured rodents representing 16 species, corresponding to an overall rodent capture rate of 6.75% (3,847/56,979). The predominant rodent species were *Rattus losea* (losea vole) (41.46%), *Rattus tanezumi* (oriental house rat) (24.64%), *Niviventer fulvescens* (chestnut white-bellied rat) (15.44%), and *Rattus norvegicus* (brown rat) (6.97%) (Figure 10).

**Figure 11:**
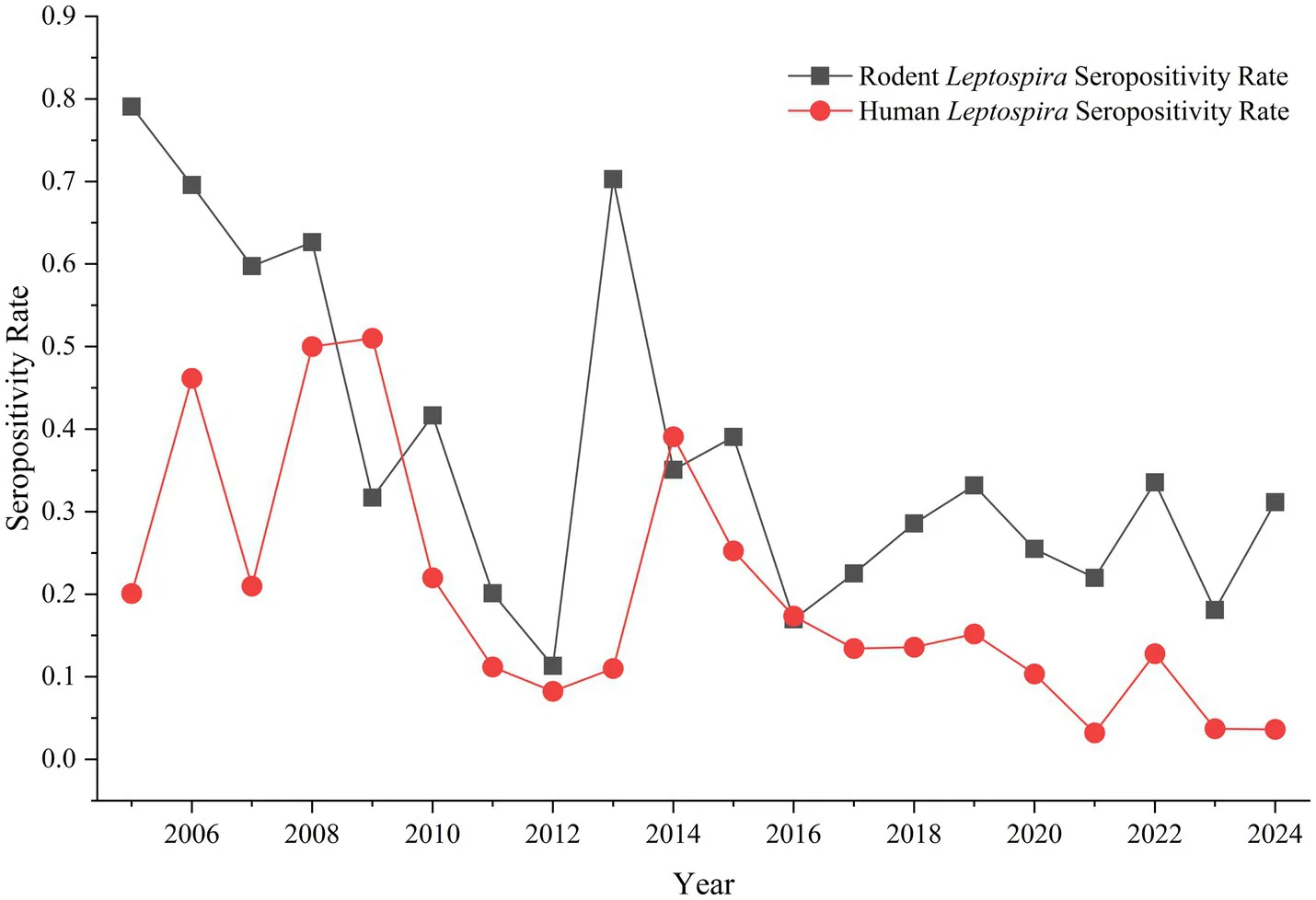
Seropositivity rates of *Leptospira* antibodies in rodents and healthy humans at surveillance sites in Fujian Province, 2005–2024.

Serum samples from rodents were tested for leptospiral antibodies. The seropositivity rates ranged from 11.32 to 78.82%, with an overall mean seropositivity rate of 32.89% (Figure 11). The highest seropositivity rate was observed in 2005 (78.82%), followed by a decline to a nadir of 11.32% in 2012. Subsequently, the rate increased again, reaching a second-highest value in 2013 (70.27%), before gradually declining thereafter. The chi-square test for trend demonstrated a significant linear trend in rodent seropositivity rates from 2005 to 2024 (*χ*^2^_trend = 79.97, *p* < 0.001).

**Figure 12:**
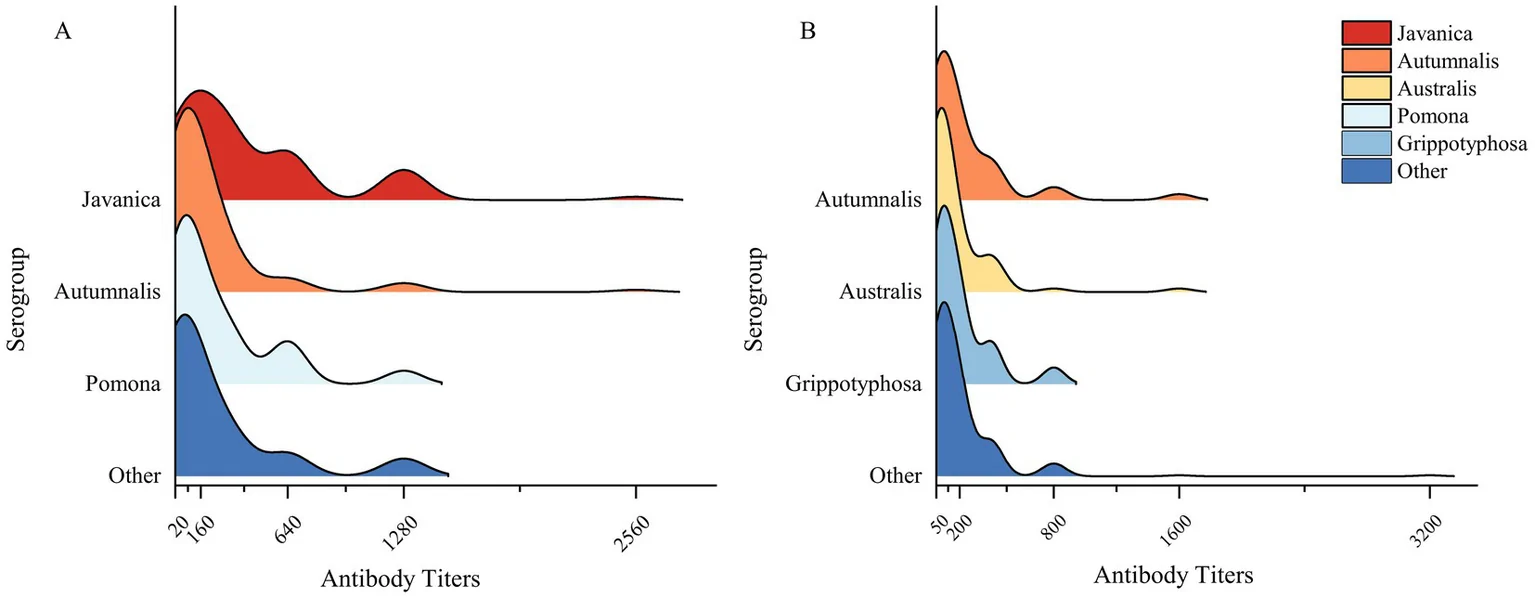
Distribution of antibody titers against different *Leptospira* serogroups in rodents and healthy humans at leptospirosis surveillance sites in Fujian Province, 2005–2024: **(A)** antibody titers of major leptospiral serogroups in rodent serum; **(B)** antibody titers of major leptospiral serogroups in serum from healthy individuals.

**Figure 13:**
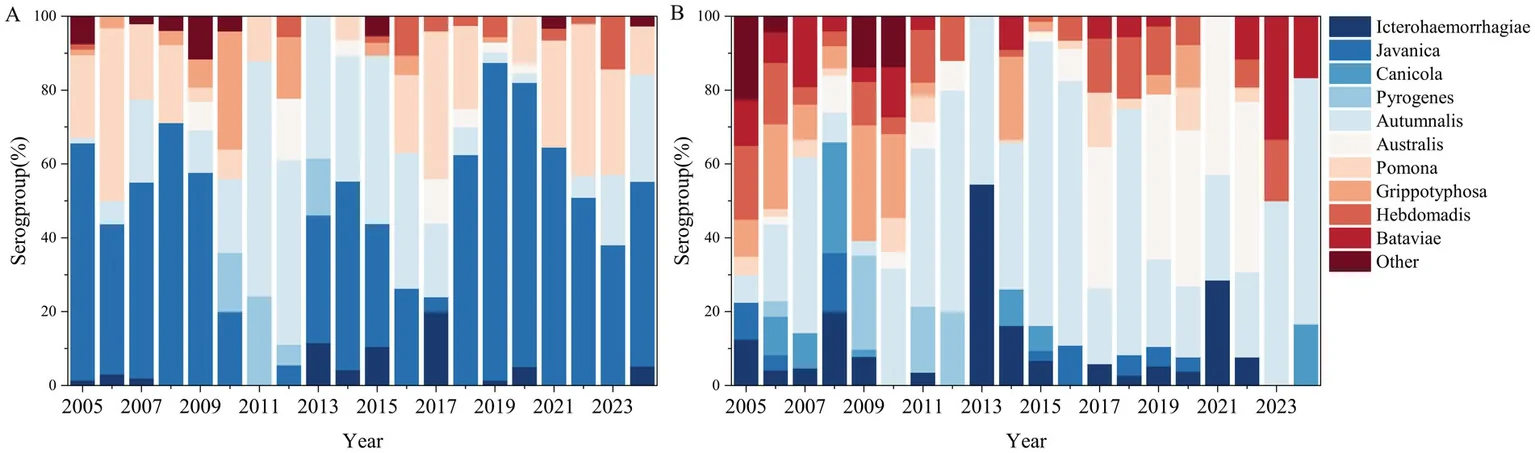
Serogroup distribution of *Leptospira*-positive sera from rodents and healthy humans at surveillance sites in Fujian Province, 2005–2024. **(A)** Leptospiral serogroups identified in rodent sera; **(B)** leptospiral serogroups identified in sera from healthy populations.

Antibodies against *Leptospira* were detected in sera from 12 rodent species. The highest seropositivity rates were observed in *Rattus losea* (40.99%) and *Rattus tanezumi* (36.86%). The geometric mean titer (GMT) among all antibody-positive samples was 1:158 (95% *CI*: 1:146–1:172), with titers ranging from 1:20 to 1:2560. The most common antibody titers were 1:80, 1:160, and 1:320, which together accounted for 54.20% of all positive samples. At the serogroup level, the overall titer distribution showed a similar pattern (Figure 12). With respect to serogroup distribution, the predominant *Leptospira* serogroup detected in host animals was Javanica (50.52%), followed by Autumnalis (18.23%) and Pomona (16.93%) (Figure 13). At the rodent species level, *Rattus losea* was predominantly infected with the Javanica serogroup, followed by Pomona and Autumnalis. Similarly, *Rattus tanezumi* primarily harbored the Javanica serogroup, with Autumnalis and Pomona detected at lower frequencies.

In the two counties under long-term surveillance (Changtai District and Wuping County), rodent seropositivity rates exhibited significant declining trends (*χ*^2^_trend = 68.23, *p* < 0.001; *χ*^2^_trend = 38.81, *p* < 0.001). The mean seropositivity rate was 46.93% in Changtai District and 39.77% in Wuping County. The predominant *Leptospira* serogroup in both areas was Javanica, accounting for 43.9% in Changtai District and 60.2% in Wuping County. Regarding antibody titers, from 2005 to 2018, the GMT of *Leptospira* antibodies in rodents from Changtai District ranged from 1:80 to 1:356, peaking at 1:356 in 2009, followed by an overall declining trend. In Wuping County, the GMT ranged from 1:28 to 1:390 during 2011–2024. GMT levels remained relatively low during 2011–2012, reached a peak of 1:390 in 2014, and subsequently stabilized between 2016 and 2024.

#### 3.4.2 Healthy population surveillance

From 2005 to 2024, a total of 3,964 serum samples were collected from healthy individuals at surveillance sites. Among these, 706 samples tested positive for leptospiral antibodies, with seropositivity rates ranging from 3.21 to 51.00%, and an average seropositivity rate of 17.81% (Figure 11). A chi-square test for trend revealed a significant overall decline in seropositivity over time (*χ*^2^_trend = 143.42, *p* < 0.001). With respect to serogroup distribution, antibodies against *Leptospira* belonging to 13 serogroups and 13 serovars were detected. The predominant circulating serogroup was Autumnalis (37.11%), followed by Grippotyphosa (10.48%) and Australis (10.34%) (Figure 13). The Autumnalis serogroup was detected in all surveillance years. During 2005–2014, the predominant serogroups were Autumnalis, Grippotyphosa and Icterohaemorrhagiae, whereas from 2015 onwards, Autumnalis, Australis, and Hebdomadis became dominant. Fisher’s exact test indicated statistically significant interannual differences in serogroup composition among healthy individuals at surveillance sites (*p* < 0.001). Most seropositive individuals exhibited antibody titers of 1:50, 1:100, or 1:200, accounting for 71.95% of positive samples, while eight individuals had antibody titers ≥1:1600. When stratified by serogroup, the overall titer distribution patterns were broadly consistent (Figure 12).

The overall seropositivity rates among healthy populations under long-term surveillance were 27.97% in Changtai District and 19.18% in Wuping County. In both areas, seropositivity rates exhibited significant declining trends over time (*χ*^2^_trend = 8.25, *p* < 0.05; *χ*^2^_trend = 8.72, *p* < 0.05). In Changtai District, seroprevalence gradually increased from 2005 to 2009, followed by a gradual decline. A secondary rise occurred beginning in 2013, reaching a peak in 2014, after which the rate decreased again. In Wuping County, seroprevalence showed a gradual increase from 2011, peaked in 2018, and subsequently declined. The GMT in Changtai District was 1:142.6 (95% CI: 1:131.7–1:154.4), exhibiting an overall downward trend, whereas the GMT in Wuping County was 1:120.0 (95% CI: 1:102.8–1:140.0) and remained relatively stable over the study period. With respect to serogroup distribution, the predominant *Leptospira* serogroup in Wuping County was Autumnalis. In Changtai District, antibodies against *Leptospira* belonging to 13 serogroups were detected, with Autumnalis (*n* = 99), Grippotyphosa (*n* = 64), and Icterohaemorrhagiae (n = 41) being the most prevalent. During 2005–2007, the dominant serogroups in Changtai District were Hebdomadis, Grippotyphosa, and Autumnalis. In 2008, Canicola emerged as the predominant serogroup, followed by Grippotyphosa in 2009. From 2011 to 2018, Autumnalis consistently remained the dominant serogroup.

#### 3.4.3 Correlation between human incidence rate, host animal seropositivity rates, and healthy population seropositivity rates

Spearman’s rank correlation analysis was conducted to assess the relationships among the incidence rate at monitoring sites, the seropositivity rate in reservoir animals, and the seropositivity rate in the healthy population. The incidence rate at surveillance sites was not significantly correlated with the seropositivity rate in reservoir animals (*p* = 0.051) or with that in the healthy population (*p* = 0.539). In contrast, a statistically significant positive correlation was observed between the seropositivity rates in reservoir animals and the healthy population (*ρ* = 0.568, *p* = 0.009).

Further stratified analyses conducted separately for Changtai District and Wuping County showed no statistically significant correlations among the three indicators in either area (all *p* > 0.05).

## 4 Discussion

The incidence of leptospirosis in Fujian Province showed a continuous decline from 2004 to 2024, with the exception of a few isolated years. This pattern was consistent with the overall epidemiological trend in China and may be attributable to rapid economic development, improvements in ecological and environmental conditions, strengthened rodent control programs, and increasing levels of agricultural mechanization (6, 12). However, as a re-emerging zoonosis of global concern, leptospirosis continues to pose a public health challenge that necessitates continued vigilance. The global epidemiological landscape is characterized by pronounced spatial heterogeneity; the disease remains disproportionately prevalent in tropical and subtropical regions (13, 14), such as Brazil (15), Sri Lanka (16), and Fiji (17, 18). In these locales, conducive climatic conditions-notably high temperatures and heavy precipitation-facilitate the environmental survival and transmission of pathogenic *Leptospira*, resulting in substantial morbidity and economic losses. This underscores the decisive influence of ecological and environmental drivers in shaping regional epidemic patterns. Furthermore, although incidence rates in southern China, including Fujian Province, have declined substantially from historical peaks, more than 99% of national cases remain clustered in the south, reflecting a stark and persistent north–south disparity (6).

In Fujian Province, July to October constitutes the annual peak season for leptospirosis incidence. This seasonal pattern is closely associated with increased precipitation during this period, driven by meteorological factors such as typhoons and heavy rainfall. Frequent rainfall can wash *Leptospira*-contaminated soil into rivers, ponds, and irrigation systems, leading to contamination of both stagnant and flowing water bodies and substantially increasing the risk of human exposure (19). In addition, this period coincides with intensive agricultural activities, during which farmers are more frequently exposed to contaminated water and soil, further increasing the risk of infection.

From 2004 to 2024, the median age of leptospirosis cases in Fujian Province increased by approximately 20 years, with infections predominantly occurring among individuals aged 30–69 years. Farmers consistently represented the largest occupational group affected, a finding consistent with previous reports by Liu et al. (20), which indicated that middle-aged and older adults gradually replaced young adults as the primary affected population in China. This demographic shift is likely related to rapid socioeconomic development and large-scale migration of younger adults to urban areas. The remaining agricultural workforce is increasingly composed of older residents, whose routine farming activities—such as rice transplanting, field management, and harvesting—entail frequent exposure to waterlogged fields and soil environments contaminated with *Leptospira*, thereby elevating infection risk (6, 21). In addition, the higher proportion of male cases observed in this study is consistent with reports from multiple regions and countries (3, 22–24). This pattern may be attributed to greater male participation in outdoor and water-related agricultural work, resulting in more frequent exposure to potentially contaminated environments.

Global spatial autocorrelation analysis demonstrated that the distribution of leptospirosis cases in Fujian Province from 2004 to 2024 was non-random, indicating significant spatial clustering. Local spatial autocorrelation analysis identified a high-high clustering area in Songxi County, where a predominantly agricultural economy and intensive farming activities may increase opportunities for exposure to *Leptospira*-contaminated water and soil. These findings indicate that Songxi County should be considered a priority area for enhanced leptospirosis surveillance and targeted prevention and control measures. In contrast, cities in southern Fujian, including Zhangzhou, Xiamen, Quanzhou, and Putian, are predominantly coastal and exhibit higher levels of socioeconomic development. Their industrial structures are largely driven by manufacturing, service industries, and tourism, with a comparatively smaller proportion of agricultural production. Rapid urbanization and land-use transformation have reduced cultivated land area and shifted occupational patterns away from traditional water-intensive farming activities, potentially decreasing population exposure to contaminated environments and partially explaining the relatively lower incidence observed in these areas.

Spatiotemporal cluster analysis further revealed that reported leptospirosis cases in Fujian Province from 2005 to 2024 formed six significant clusters, which were primarily distributed in Wuping County of Longyan City and parts of Nanping, Sanming, and Ningde. Among these, Wuping County constituted the most likely spatio-temporal cluster, with a clustering window extending from June 2005 to August 2014, and cases predominantly occurring between July and September. This period coincides with peak agricultural activity as well as high temperature and rainfall, conditions favorable for the environmental survival and transmission of *Leptospira*. In addition, Wuping County has historically been recognized as a leptospirosis-endemic area in Fujian Province. The persistence of significant clustering over the past two decades suggests sustained transmission risk in this region. These findings provide useful information for prioritizing surveillance resources and implementing season-specific prevention and control strategies in high-risk areas.

The occurrence of human leptospirosis is influenced by multiple factors, among which the density of animal hosts and their *Leptospira* carriage rates are particularly important (25). Rodents, as the principal reservoir hosts, play a critical role in maintaining and transmitting of the pathogen. Surveillance data from Fujian Province indicate an average rodent capture rate of 6.75%, with *Rattus losea* and *Rattus tanezumi* identified as the predominant species. This species composition differs markedly from that reported in other regions of China. For example, in Shanxi Province, the predominant species were *Apodemus agrarius* (striped field mouse) and *Niviventer confucianus* (Confucian niviventer), with the latter exhibiting the highest *Leptospira* detection rate (26). In Jiangxi Province, monitoring conducted between 2016 and 2018 reported an average rodent density of 6.76%, with *Apodemus agrarius* and *Rattus losea* as the predominant species (27). Overall, although rodent abundance indicators may vary across regions, the composition of dominant rodent species differs substantially, indicating pronounced geographical heterogeneity in the natural foci of leptospirosis in China. These interregional differences in host composition may influence *Leptospira* carriage rates, serogroup distribution, and transmission dynamics, thereby contributing to regional variation in leptospirosis epidemiology.

Although human leptospirosis incidence, host animal seropositivity rates, and seropositivity rates in healthy populations have all shown overall declining trends, seropositivity remained relatively high, particularly among rodents. Rodent seropositivity rates ranged from 11.32 to 78.82%, with annual rates consistently exceeding 10.00%. Notably, rodent seropositivity decreased to its lowest level in 2012 but increased sharply to approximately 70% in 2013 before declining again in 2014. This fluctuation may be partly attributable to regional sampling differences, as surveillance in 2013 was conducted primarily in Changtai County. In addition, because additional surveillance counties were selected annually according to the provincial surveillance program, differences in surveillance locations may also have contributed to the observed year-to-year variation.

The predominant serogroups detected in rodent were Javanica, Autumnalis, and Pomona, with the highest positivity observed in *Rattus losea* and *Rattus tanezumi*. Similar patterns of high rodent seroprevalence have been reported in multiple regions worldwide. In the central northern region of Algeria, MAT testing of 100 rats revealed a seropositivity rate of 43%, predominantly against Icterohaemorrhagiae (28). In Brazil, a serological study involving 122 rats reported a seropositivity rate of 72%, with Icterohaemorrhagiae and Bataviae as the main serotypes (29). Likewise, a MAT-based survey of 122 rodents from Guayaquil City in Ecuador, conducted between 2017 and 2020, demonstrated a seropositivity rate of 83.6%, with Saxkoebing as the predominant serovar (30). These findings indicate marked global heterogeneity in leptospiral antibody prevalence and dominant serogroups/serovars among rodent populations. In the present study, leptospiral serogroups detected in healthy human populations were mainly Autumnalis, Grippotyphosa, and Australis, showing distribution patterns that differed from those identified in reservoir animals during the same period. This discrepancy suggests that the circulation and transmission dynamics of *Leptospira* are not fully concordant between animal reservoirs and human populations. Human infection patterns are likely shaped by the combined effects of multiple reservoir hosts, environmental exposure pathways, and serogroup-specific host adaptation. Previous studies have demonstrated marked differences in serovar distribution among host species, with certain serovars detected exclusively or preferentially in specific animal hosts (31). Moreover, serovars identified in human infections do not necessarily correspond closely to those detected in a single reservoir species. Therefore, these findings highlight the importance of systematic multi-host investigations to identify key reservoir hosts in local transmission cycles and to better elucidate their respective roles in human leptospirosis. Such investigations provide valuable information on *Leptospira* ecology, reservoir host dynamics, and pathogen circulation. However, the extent to which integrated reservoir surveillance can predict human disease occurrence remains to be further evaluated.

The present study showed that the seroprevalence of leptospiral antibodies in healthy populations exhibited an overall declining trend and remained at relatively low levels in recent years, with most positive samples characterized by low antibody titers. This pattern suggests relatively limited recent exposure or infection in the general population. Further analysis revealed a positive correlation between temporal changes in antibody seropositivity among healthy individuals and corresponding seropositivity rates in rodents, suggesting that human exposure to *Leptospira* is associated with infection levels in reservoir hosts. However, neither rodent nor human seropositivity was significantly associated with reported human incidence in the present study, indicating that reservoir serological indicators alone may have limited value as predictors of human disease occurrence. Nevertheless, continuous monitoring of reservoir hosts provides important information on *Leptospira* circulation, including the geographic distribution of pathogen activity, reservoir species composition, and predominant serogroups. Such information is essential for identifying potential risk areas and guiding targeted prevention and control strategies.

One possible explanation is that routine rodent surveillance in Fujian has historically focused primarily on wild or peri-natural environments. With rapid urbanization and changes in human living environments, peridomestic rodents may play an increasingly important role in the transmission of *Leptospira* (32). Therefore, whether current surveillance strategies adequately capture contemporary transmission risks deserves further evaluation, and future surveillance may benefit from a more balanced inclusion of both wild and peridomestic rodent populations. It should also be noted that leptospiral antibodies may decline over time, and seropositivity does not necessarily indicate long-lasting protective immunity (33). Therefore, even when overall population seropositivity is low, localized outbreaks may still occur if environmental exposure increases or infection prevalence among reservoir hosts rises. From a One Health perspective (34), our findings support the importance of integrating surveillance data from human cases, healthy populations, and reservoir hosts to better understand the epidemiology of leptospirosis. Although reservoir surveillance alone was not significantly associated with human incidence in this study, combining information from these surveillance components may provide a more comprehensive understanding of pathogen circulation and human exposure, which could help inform prevention and control efforts.

This study has several limitations. First, during the compilation of long-term surveillance data, some historical records were incomplete or missing. For example, detailed case information for 2004 was unavailable, which precluded the inclusion of data from that year in the spatiotemporal scanning analysis. In addition, serum samples collected in 2015 recorded only qualitative results (positive or negative), without detailed serogroup identification or antibody titer measurements. Consequently, these data could be used only to describe overall seropositivity and were not suitable for in-depth analyses of serogroup distribution or antibody level dynamics. Second, host animal species identification relied solely on morphological characteristics, which limited the verification of historical records and may have introduced misclassification bias. Third, detailed microbiological and serological information from reported human cases, including infecting serogroups or bacterial isolates, was unavailable. This limited our ability to directly compare circulating *Leptospira* serogroups between reservoir hosts and human cases and to better understand transmission links. Moreover, although MAT is the reference serological method for leptospirosis surveillance, cross-reactivity among different *Leptospira* serogroups may affect serogroup assignment, particularly in samples reacting with multiple serogroups (35). Therefore, the serogroup distribution observed in this study should be interpreted with caution, and confirmation by bacterial isolation or molecular typing would provide more definitive evidence.

## 5 Conclusion

From 2004 to 2024, the incidence of leptospirosis in Fujian Province showed a overall declining trend, accompanied by marked geographic clustering and clear seasonal patterns. Cases were primarily concentrated in northern and north-central Fujian, and the affected population gradually shifted toward older age groups. Surveillance data identified *Rattus losea* and *Rattus tanezumi* as the principal rodent species and important reservoir hosts of *Leptospira* in Fujian Province.

Although leptospiral antibody seroprevalence in rodents declined over time, it remained relatively high overall, with Javanica, Autumnalis, and Pomona as the main serogroups. Similarly, antibody seroprevalence among healthy populations also decreased, although the serogroup distribution differed from that observed in reservoir hosts, with Autumnalis predominating in humans. These findings indicate that *Leptospira* circulation differs between reservoir hosts and human populations, highlighting the complexity of local transmission dynamics. Long-term surveillance of human cases, healthy populations, and reservoir hosts provides complementary information for understanding the epidemiology of leptospirosis. Given the changing epidemiological context associated with urbanization, future surveillance strategies should continue to be evaluated and optimized to better characterize transmission patterns and support evidence-based prevention and control efforts.

## Data Availability

The raw data supporting the conclusions of this article will be made available by the authors, without undue reservation.

## Supplementary material

SUPPLEMENTARY FIGURE S1 Comparison of Leptospirosis incidence rates by stage (2004–2024) across cities.
